# A Microfluidic Chip-Based Integrated Device Combining Aerosol Sampling and LAMP–CRISPR Detection for Airborne Virus Surveillance

**DOI:** 10.3390/bios15080475

**Published:** 2025-07-23

**Authors:** Anlan Zhang, Yuqing Chang, Wen Li, Yuanbao Zhang, Yuqian Wang, Haohan Xie, Tao Zuo, Yu Zhang, Jiyu Xi, Xin Wu, Zewen Wei, Rui Chen

**Affiliations:** 1 School of Medical Technology, Beijing Institute of Technology, Beijing 100081, China; 3120225711@bit.edu.cn (A.Z.); lwhry123@163.com (W.L.); 3120246300@bit.edu.cn (Y.Z.); 15391166971@163.com (J.X.); 3120235941@bit.edu.cn (X.W.); 2 Beijing Key Laboratory of Occupational Safety and Health, Institute of Urban Safety and Environmental Science, Beijing Academy of Science and Technology, Beijing 100054, China; m18813070133@163.com (Y.C.); zhangyuanbao2013@163.com (Y.Z.); wangyuqian1314@163.com (Y.W.); xiehaohan@iuse.ac.cn (H.X.); zuotao23@mails.ucas.ac.cn (T.Z.)

**Keywords:** airborne virus, bioaerosol, loop-mediated isothermal amplification, CRISPR/Cas12a, microfluidic chip, integrated device

## Abstract

Detecting airborne viruses using an integrated aerosol sampling detection device is of great significance in epidemic prevention and control. Most of the applicable aerosol samplers have a flow rate of less than 1000 L/min, which is insufficient for application in large public spaces. Recent research, on the other hand, has revealed the advantages of microfluidic chip-based LAMP–CRISPR in airborne virus detection; however, this promising detection method has yet to be integrated with an aerosol sampler. Herein, we present an aerosol sampling and microfluidic chip-based detection (ASMD) device that couples a high-flow-rate aerosol sampling (HFAS) system with a microfluidic LAMP–CRISPR detection (MLCD) chip for surveilling airborne viruses, as represented by SARS-CoV-2. The HFAS system achieved a 6912 L/min flow rate while retaining a satisfactory collection efficiency, and achieved an enrichment ratio of 1.93 × 10^7^ that facilitated subsequent detection by the MLCD chip. The MLCD chip integrates the whole LAMP–CRISPR procedure into a single chip and is compatible with the HFAS system. Environmental detection experiments show the feasibility of the ASMD device for aerosol sampling and detection. Our ASMD device is a promising tool for large space aerosol detection for airborne virus surveillance.

## 1. Introduction

Aerosols are recognized as a primary transmission route for the severe acute respiratory syndrome coronavirus 2 (SARS-CoV-2) [1], and a study showed that the virus can remain viable and infectious in aerosols for several hours [2]. Therefore, detecting SARS-CoV-2 in aerosols is critical for controlling the coronavirus disease 2019 (COVID-19), which is still impacting countries worldwide, with the World Health Organization reporting 23,164 new confirmed cases from 7 April to 4 May 2025 [3]. A device that combines aerosol sampling and SARS-CoV-2 detection offers a promising solution for detecting SARS-CoV-2 aerosols.

Previous studies used aerosol samplers such as filters [4,5,6,7,8,9], electrostatic precipitators [10,11,12,13], cyclones [14], impactors [15], and impingers [15,16,17] to collect aerosols from the air for further analysis. Some studies integrated the samplers with strategies including pre-charging [18,19,20], turbulent deposition [21,22,23,24,25], and condensation growth [26,27,28,29] to improve the particle collection efficiency. However, since collection efficiency decreases as the air sampling flow rate increases, most of the applicable aerosol samplers have a flow rate of less than 1000 L/min [30,31,32,33]. Such relatively low flow rates limit the sampler’s application scenarios and are insufficient for rapid detection in large public spaces, since a 1000 L/min sampler takes more than 16 h to assay the total air in a 1000 m^3^ space. Therefore, there is a significant need for aerosol samplers with higher flow rates and improved collection efficiencies in order to extend the applications to large-volume places.

Recent studies, on the other hand, coupled aerosol samplers with biosensors, including electrochemical and optical sensors, to detect pathogens in collected aerosols. Puthussery et al. [31] integrated a wet cyclone air sampler with a nanobody-based micro-immunoelectrode biosensor to detect SARS-CoV-2 aerosols. Qiu et al. [8] developed a condensation growth-assisted aerosol collecting and plasmonic photothermal sensing system for SARS-CoV-2 aerosol analysis. However, the fabrication of such biosensors requires the use of specialized materials such as nanobodies and gold nanoislands, which raises costs and limits their practical application. Xiong et al. [34] constructed an aerosol SARS-CoV-2 sampling/monitoring system, which includes a filter-based aerosol sampler and a loop-mediated isothermal nucleic acid amplification (LAMP)-based rotating microfluidic fluorescence detection chip. Although being highly sensitive, LAMP detection is prone to false-positive results, because numerous and large-sized primers (30–40 bases for FIP and BIP) can self-hybridize and produce self-amplified products without templates [35]. The combination of LAMP with clustered regularly interspaced short palindromic repeats (CRISPR) provides a solution to this limitation, owing to the highly specific cleavage of target sequences [36]. Recent studies have demonstrated that LAMP–CRISPR has the potential to detect SARS-CoV-2 [37,38,39,40,41,42,43]. These studies utilized microfluidic chips, which have exhibited great potential in automated rapid detection [40,41], to minimize sample and reagent consumption and achieve sample-in–result-out detection. However, due to the technical challenges in aerosol sampling and integration, no known studies have integrated LAMP–CRISPR detection with an aerosol sampler.

Here, we present an aerosol sampling and microfluidic chip-based detection (ASMD) device that couples a high-flow-rate aerosol sampling (HFAS) system with a microfluidic LAMP–CRISPR detection (MLCD) chip to detect SARS-CoV-2 from ambient aerosols in 85 min (45 min aerosol sampling and 40 min LAMP–CRISPR detection). The HFAS system uses electrostatic sampling, air–liquid interface sampling (aerosol-to-hydrosol sampling), and magnetic bead enrichment to achieve at least a 74.6% collection efficiency for aerosol particles ranging from 10 to 1000 nm at flow rates of up to 6912 L/min. Such a high flow rate and satisfactory collection efficiency of the system compensate for the comparatively low flow rates of conventional air samplers, making it suitable for long-term routine surveillance of SARS-CoV-2 aerosols in large indoor spaces such as hospitals, schools, shopping malls, and hotel buildings. The specially designed MLCD chip integrates RNA loading, LAMP amplification, and CRISPR detection into a single chip and is compatible with the HFAS system, enabling the development of an all-in-one aerosol sampling–detection device. More specifically, the chip performs the LAMP reaction to yield highly specific amplification products of the targeted RNA, which contain fluorescence reporter probes made up of single-stranded deoxyribonucleic acids (ssDNAs) with a fluorophore on one end and a quencher on the other [36]. Then, the chip utilizes a CRISPR-associated protein, CRISPR/Cas12a, which specifically targets the RNA sequence and performs ssDNA cleavage, liberating the fluorophore and producing a fluorescent signal for detection [36]. Furthermore, the MLCD chip’s multi-unit design enables the simultaneous detection of multiple variants. The all-in-one ASMD device realizes air-in–result-out detection of SARS-CoV-2 aerosols, simplifying the aerosol detection process, eliminating human error, avoiding sample or reagent loss, and improving detection repeatability. Environmental aerosol detection experiments demonstrate that the ASMD device can be used to detect SARS-CoV-2 aerosols in large public spaces on-site. The primary limitation of the device lies in its current inability to determine the concentrations of the detected viruses. This is because the fluorescence intensities did not exhibit any significant differences across the various concentrations we tested. However, we believe that with further improvement, such as optimizing the LAMP cycle number and improving camera resolution, this limitation can be overcome to provide more specific information on the detected aerosols.

## 2. Materials and Methods

### 2.1. Fabrication Process of the MLCD Chip

As shown in Figure S2, two polydimethylsiloxane (PDMS) layers and a flexible membrane layer were separately fabricated, and then the membrane layer and the PDMS liquid channel layer were sequentially bonded to the PDMS pneumatic control layer. The PDMS layers were fabricated by casting PDMS onto SU-8 molds, which were coated and patterned on silicon wafers. The membrane layer was fabricated by spinning SU-8 onto a silicon wafer.

### 2.2. Theoretical Calculation of the Aerosol Sampling System

Effective design for aerosol sampling in large spaces:

The sampling coefficient, denoted as *κ*, is defined as the ratio of the effectively sampled air volume (*V_eff_*) to the total spatial volume (*V_tot_*), weighted by the sampler’s capture efficiency (η) [42]. Therefore, *κ* is calculated as follows:

*κ* = (*V_eff_*/*V_tot_*) × η (1)

*κ* = 1 represents ideal conditions where 100% of the air volume is sampled with a 100% capture efficiency. Statistically representative sampling is generally considered achievable when *κ* is greater than or equal to 0.25, which can guarantee reliable data regarding aerosol concentration and distribution [42]. In high-precision applications, such as pathogen monitoring, a more stringent criterion of *κ* ≥ 0.5 is recommended [42].

To illustrate, consider a room with a total volume (*V_tot_*) of 240 m^3^, similar to a typical school classroom. If a 30-min pathogen monitoring sampling is needed, it is advisable to employ at least four sampling devices, each with a flow rate of 1000 L/min, using a multi-grid sampling and detection approach. This setup can help meet the sampling requirements in such a space. For the sake of simplicity, the sampler’s capture efficiency was set to 1 in this calculation.

Minimum airflow rate calculation:

According to Equation (1), the airflow rate *Q* can be calculated as follows:

*Q* = (*V*/*t)* × *κ*, (2)

where *V* is the room volume and *t* is the sampling time. Thus, for a *V* = 60 m^3^ room, to achieve an effective sampling (*κ* = 0.5) in the room within *t* = 30 min, the minimum required flow rate *Q* is calculated as follows:

*Q* = (*V*/*t)* × *κ* = 1000 L/min (3)

Breakdown voltage estimation:

Tests revealed that when the distance *d* between two electrode plates is less than 3 m, the breakdown voltage *V* has a linear relationship with *d*, and the breakdown voltage per unit distance is defined as breakdown electric intensity. Air breakdown electric intensity can be estimated at 30 kV/cm according to empirical data [44]. In this study, the maximum voltage was set to 50 kV at a distance of 25 cm, which is safe from electrical breakdown of air.

Corona pin selection and setup:

Compared with spiral, straight, and round-shaped corona pins, palm-shaped or serrated corona pins show better electrical properties. Hence, palm-shaped and serrated corona pins were chosen for the pre-charging zone. The corona pin was placed in the middle of the pre-charging zone and connected to the high-voltage DC power supply. The shell of the pre-charging zone was connected to the ground. The pin’s length was 20 cm.

### 2.3. Collection Efficiency Tests

Collection efficiency tests for 10–1000 nm particles under various voltages and flow rates were performed in a 6 m × 3.5 m × 3 m room with an air volume of 6.3 × 10^4^ L. A P-Trak 8525 device was used to measure the concentrations of 10–1000 nm particles in the air. First, the initial particle concentration in the room (*C_in_*) was measured using the P-Trak 8525 device. The room was then closed, and the HFAS sampler was switched on to perform air sampling. The particle concentration at the sampler’s outlet (*C_out_*) was measured immediately. It should be noted that the room was closed, and the measurement was performed immediately to minimize the sampler’s influence on the particle concentration in the air. The room was then opened, allowing the air particle concentration to recover. Lastly, the pre-charging voltage, collecting voltage, and fan flow rate were adjusted, and the test was repeated. The collection efficiencies were calculated using the following equation:

*η* = (*C_in_* − *C_out_*)/*C_in_*, (4)

P-Trak 8525 (TSI Incorporated, Shoreview, MN, USA) is a portable instrument that detects and counts ultrafine particles (smaller than 1 µm); it consumes alcohol to grow microscopic particles in the air into larger droplets that are easier to detect and count [45]. Although the instrument can produce errors due to factors such as limited collection efficiency, it is believed that the error is minimal and acceptable as long as the operating conditions meet the requirement (0–38 °C, 100% reagent grade isopropyl, etc.) [45].

### 2.4. RNA Sample Preparation

First, PCR was performed to amplify a 493 nt S-gene fragment from an ordered plasmid (Sangon Biotech, Shanghai, China) containing SARS-CoV-2 S-gene (nt 21563-nt 25384; GenBank: NC_045512.2). The amplification mixture consisted of 12.5 μL Q5 Hot Start High-Fidelity 2X Master Mix (New England Biolabs, Ipswich, MA, USA), 1.25 μL 10 μM forward primer, 1.25 μL 10 μM reverse primer, 1 μL SARS-CoV-2 S-gene plasmid, and 9 μL ddH_2_O. Table S2 shows the primer sequences. For PCR amplification, the mixture was first denatured at 98 °C for 30 s. Then it was subjected to 35 cycles of 5 s denaturation at 98 °C, 10 s annealing at 68 °C, and 20 s extension at 72 °C. Lastly, it was heated at 72 °C for 120 s for additional extension, then cooled down and stored at 4 °C.

Next, the amplification products were verified using 1.2% agarose gel electrophoresis. The amplified DNA sample was mixed with 6 × loading buffer in a 5:1 volume ratio. DNA markers and the mixture were sequentially added to the gel pores. Electrophoresis was performed at 100 V for 30 min.

After electrophoresis verification of the amplified DNA products, the products were retrieved using the gel recovery kit (Science Tool, Hong Kong, China) according to the manufacturer’s instructions. Then, 1 μg of the DNAs was used as a template to synthesize RNAs using the HiScribe T7 High Yield RNA Synthesis Kit (New England Biolabs, Ipswich, MA, USA). After that, the RNA solution was cooled down and 1 μL of DNase I (New England Biolabs, Ipswich, MA, USA) and 10 μL of DNase I Reaction Buffer were added. The solution was diluted to 100 μL using purified water, and the reaction was incubated at 37 °C for 10 min to remove the DNA templates.

Finally, the obtained RNAs were purified through the following steps. First, the bottle containing RNAClean XP (Beckman Coulter, Brea, CA, USA) was shaken to resuspend the magnetic beads, and RNAClean XP was added to the RNA solution at a ratio of 1.8:1 (XP: solution). The mixture was pipetted to mix the magnetic beads with the target RNAs, then incubated at room temperature for 5 min. Second, the tube with the mixture was placed on a magnetic grate to separate the RNA-binding beads from the solution. Third, 500–1000 μL of 70% ethanol was added to the beads and the reaction was incubated at room temperature for 30 s. The ethanol was discarded and the washing step was repeated three times, after which the RNA-binding beads were air-dried by opening the tube. Fourth, the purified RNAs were eluted from the beads using enzyme-free water, and the RNA concentration was measured using a spectrophotometer. The purified RNA sample was stored at −80 °C.

### 2.5. Aerosol Sampling-Detection Experiments

Artificial aerosol experiment: RNA sample solution was diluted and then added to a K5 electric sprayer to generate aerosol particles at a spray rate of 15 mL/min. Using the ASMD device, the generated aerosols were collected at voltages of 50 kV for pre-charging and 50 kV for collecting, with a flow rate of 6912 L/min; 1 mL of RNA solution was sprayed each time at 5 min intervals to prevent excessive humidity, which could lead to electrical breakdown in the pre-charging zone. As a result, a total of 150 mL of solution was divided into 150 sprays. The sampled liquid from the multiple HFAS channels was delivered to different 15 mL centrifuge tubes.

Then, LAMP–CRISPR was performed to detect SARS-CoV-2 RNAs in the aerosol sample. First, LAMP reaction solution was prepared using a WarmStart LAMP Kit (New England Biolabs, Ipswich, MA, USA) according to the manufacturer’s instructions. The sequence information for primers, ssDNA reporter and crRNAs can be found in Tables S1 and S2. Next, 40 μL LAMP reaction solution was added to a 1.5 mL EP tube, followed by covering with 40 μL of mineral oil. Then, 20 μL of Cas12a reaction solution was added to the inner side of the tube’s cap. Then, the EP tube was heated at 65 °C for 40 min. After cooling down, the tube was shaken to mix the liquid on the cap with the underlying LAMP solution. The mixture was heated again at 37 °C for 5 min. After that, the fluorescence was observed under 480 nm blue light.

Environmental aerosol experiment: The aerosol sampling and detection procedures using the ASMD device were the same as described above, with the exception that the environmental experiment did not include aerosol generation, and the aerosol samples were directly collected from the ambient air.

### 2.6. On-Chip Detection Experiments

RNA sample detection: RNA samples of the S-gene, as well as the D614G and N501Y variants, were prepared as described. RT-LAMP reaction solution and CRISPR reaction solution were also prepared as described. The prepared reagents were injected into the MLCD chip at a flow rate of 5 μL/min using an injection pump (Biotaor Instrument, Jiaxing, China). First, RNA samples and LAMP reaction solution were injected, followed by closing the outlet ports. Then the chip was heated at 65 °C for 30 min to perform the LAMP reaction. After cooling, the chip was injected with CRISPR reaction solution and heated at 37 °C for 5 min. Finally, the produced fluorescence was observed using a fluorescence microscope. A detailed description of the experimental procedures is provided in Figure S3.

Pseudovirus sample detection: The on-chip detection experiment described above for RNA samples was repeated for the RNA sample extracted from SARS-CoV-2 pseudoviruses (Sangon Biotech, Shanghai, China).

### 2.7. Statistical Analysis

Data were presented as mean ± SD. Unpaired *t*-tests were used to compare the means of two groups. All data were analyzed by GraphPad Prism 9.5. * *p* < 0.05 was considered statistically significant.

## 3. Results

### 3.1. Combining Aerosol Sampling and Microfluidic Chip-Based LAMP–CRISPR Detection in a Multi-Module Device

**Figure 1:**
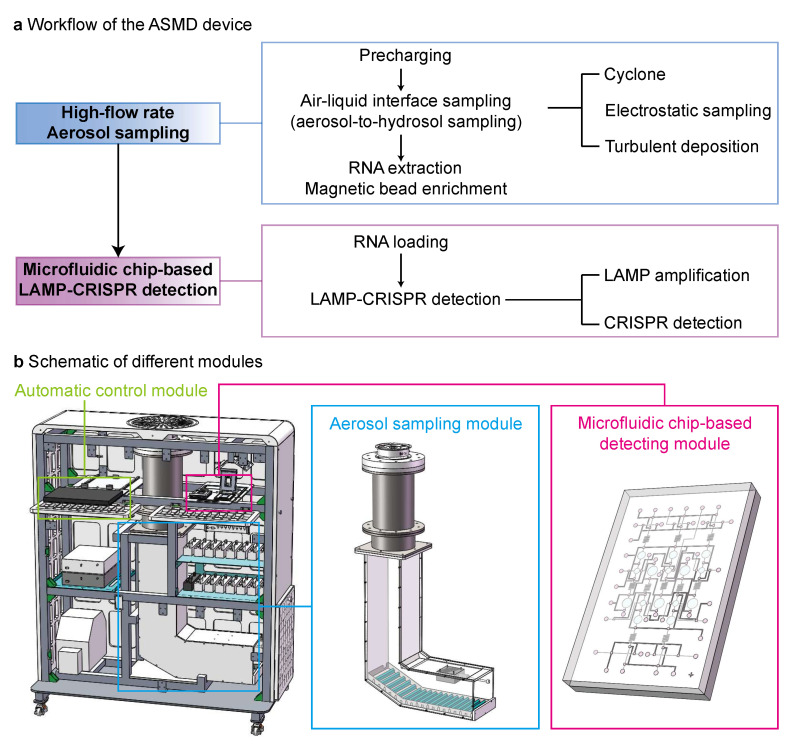
Schematic of combining high-flow-rate aerosol sampling and microfluidic chip-based LAMP–CRISPR detection for SARS-CoV-2 surveillance in an integrated ASMD device. (**a**) Workflow of the ASMD device, which consists of two steps. (**b**) Schematic of three different modules of the ASMD device.

Figure 1a illustrates the workflow of the Aerosol Sampling and Microfluidic chip-based Detection (ASMD) device, which consists of two steps. The first step is high-flow-rate aerosol sampling, which takes 45 min for up to 207 m^3^ ambient air sample analysis (6912 L/min air intake for 30 min). This step incorporates several aerosol sampling strategies including pre-charging, air–liquid interface sampling, which directly collects aerosol particles into hydrosols at the air–liquid interface by cyclone, electrostatic sampling and turbulent deposition, and RNA extraction followed by magnetic bead enrichment. The second step, microfluidic chip-based LAMP–CRISPR detection, takes 40 min. This step involves RNA loading followed by LAMP–CRISPR detection, which consists of LAMP amplification and CRISPR detection. Figure 1b shows a schematic of the device’s different modules, including an automatic control module, an aerosol sampling module, and a microfluidic chip-based detecting module.

**Figure 2:**
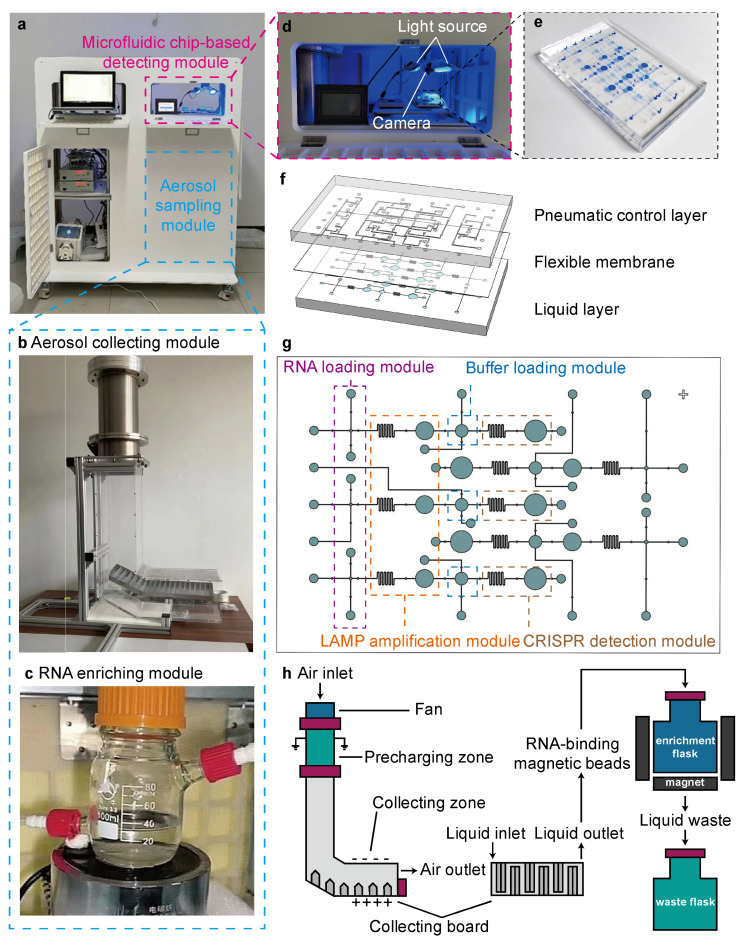
Photos and design of the ASMD device. (**a**) A photo of the ASMD device, which consists of an aerosol sampling (collecting and enriching) module and a microfluidic chip-based detection, module. (**b**) A photo of the aerosol collecting module. (**c**) A photo of the RNA enrichment module. (**d**) A photo of the microfluidic chip-based detecting module. (**e**) A photo of the MLCD chip. (**f**) Three-layer structure of the MLCD chip. (**g**) Functional modules of the MLCD chip. (**h**) Design of the HFAS system for aerosol sampling.

Figure 2a–e shows the photos of the ASMD device (Figure 2a) and its various modules, including a high-flow-rate aerosol sampling (HFAS) module, which consists of an aerosol collecting module (Figure 2b) and an RNA enrichment module (Figure 2c), as well as a detecting module (Figure 2d) based on a microfluidic LAMP–CRISPR detection (MLCD) chip (Figure 2e).

As shown in Figure 2f, the MLCD chip consists of three layers: a pneumatic control layer, a flexible membrane layer, and a liquid channel layer. Together with the flexible membrane, microvalves in the pneumatic control layer regulate the fluid flow. The liquid channel layer is designed to complete the whole process of LAMP–CRISPR detection in a single chip. As illustrated in Figure 2g, the liquid channel layer consists of five identical units for simultaneous detection of three different SARS-CoV-2 variants and two control samples. Each single unit consists of four modules: an RNA loading module, a LAMP amplification module, a buffer loading module, and a CRISPR detection module. The RNA loading module loads the RNA sample to the sample chamber of a specific volume. The LAMP amplification module has a passive mixing channel that mixes the RNA sample with LAMP reaction buffer, followed by amplification in the LAMP chamber under 65 °C. Similar to the RNA loading module, the buffer loading module loads CRISPR reaction buffer to the buffer chamber. The CRISPR detection module has another mixing channel that mixes the LAMP reaction products with CRISPR reaction buffer, while the CRISPR chamber performs CRISPR reaction under 37 °C, followed by visualized detection using a light source and a camera. The total volume of liquid sample consumption is only 0.03 μL for three parallel tests on one chip, significantly reducing the sample consumption compared to off-chip detection, which requires 1 μL for only one test. Detailed dimensions, fabrication process, and operating procedures of the MLCD chip are illustrated in Figures S1–S3, respectively. PDMS was selected for the fabrication process because it is a common material utilized for microfluidic device fabrication, owing to its excellent properties, including being cheap, easy to mold, and good for prototyping, as well as presenting optical transparency, gas permeability, biocompatibility, low autofluorescence, natural hydrophobicity, and high elasticity [46]. A block diagram of the microfluidic chip-based detection system and component details are provided in Figure S4.

Figure 2h illustrates the aerosol sampling process using the HFAS system. When the axial flow fan at the device’s inlet draws in air at a flow rate of 6912 L/min, aerosol particles in the air are pre-charged in the pre-charging zone to enhance the electric field force applied to the particles later. Next, the particles enter the collecting zone and are collected into hydrosols by electrostatic sampling under an electric field force. The collecting zone’s 90° angle generates a cyclone effect, depositing aerosols onto the pipe’s inner wall, which is equipped with triangular ridges to enhance particle collection efficiency by turbulent deposition [22]. Delivered to the collecting channel, lysis buffer containing magnetic beads lyses the virus collected, releasing target RNAs that are absorbed by the magnetic beads. The collected sample liquid, which contains RNA-binding magnetic beads, is then delivered to the enrichment flask, where the RNA beads are adsorbed by the surrounding magnets, while the remaining liquid goes to the waste flask. Finally, the target RNAs are eluted by eluant buffer to obtain the enriched RNAs for on-chip detection. Figure S5 shows detailed dimensions and structures of the aerosol sampling system. Figure S6 shows a block diagram of the HFAS system. Details on the operation of the system, consisting of 45 min aerosol sampling and 40 min on-chip detection, are provided in Figure S7.

To integrate aerosol sampling and microfluidic chip-based detection, the sample inlet of the chip is connected to the outlet of the enrichment flask, so that the entire aerosol sampling-on-chip detection process is finished in an air-in–result-out way, which simplifies the aerosol detection process, eliminates human error, avoids possible sample or reagent loss, and thus improves detection repeatability. A specially designed computer software automatically controls the integrated ASMD device (Figure S8).

### 3.2. Aerosol Sampling and On-Chip LAMP–CRISPR Detection Using the ASMD Device

**Figure 3:**
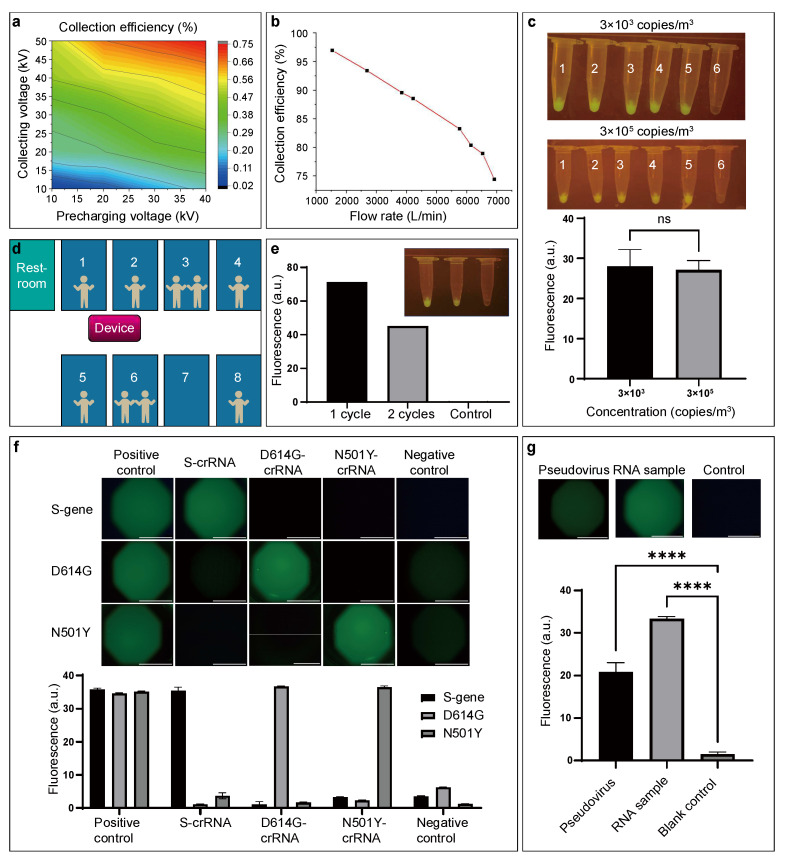
Characterization and functional validation of the MLCD chip-based ASMD device. (**a**) Particle collection efficiency in response to pre-charging voltage and collecting voltage under the flow rate of 6912 L/min. (**b**) Particle collection efficiency in response to flow rate. (**c**) Photos of aerosol generation–sampling and LAMP–CRISPR detection results and corresponding fluorescence intensity with the RNA concentration of 3 × 10^3^ and 3 × 10^5^ copies/m^3^. (**d**) Layout of the environmental detection experiment setup. (**e**) Aerosol sampling–LAMP–CRISPR detection results of the environmental detection experiment after one and two enrichment cycles, and corresponding fluorescence intensity. (**f**) Fluorescence images and fluorescence intensity of on-chip LAMP–CRISPR detection results with different RNA samples and different crRNAs. The RNA samples’ concentration is 1 × 10^9^ copies/μL. Scale bars: 100 μm. (**g**) On-chip LAMP–CRISPR detection results comparing pseudovirus samples with RNA samples. The pseudoviruses’ concentration is >1 × 10^8^ copies/mL. Scale bars: 100 μm. A.u.: arbitrary unit. **** *p* < 0.0001 (* *p* < 0.05 was considered statistically significant). (Note: We have revised Figure 3b and adjusted the maximum y value from 1 to 100.)

A critical issue faced by high-flow-rate aerosol samplers is the limited particle collection efficiency, which usually decreases as the flow rate increases. To address this problem, the HFAS system combines multiple ways of collecting aerosols and increasing efficiency. The resulting collection efficiencies under various voltages and flow rates were determined using a P-Trak 8525 device (detailed in the Section 2). As shown in Figure 3a, the collection efficiency increases as the pre-charging voltage and collecting voltage increase. Therefore, the pre-charging voltage and the collecting voltage are set at the highest feasible values of 40 kV and 50 kV, respectively, which are safe from electrical breakdown (calculated in the Methods section). Figure 3b shows the overall collection efficiency for particles within 10–1000 nm in response to an elevated flow rate between 1535 and 6912 L/min. As expected, collection efficiency is inversely related to the air sampling flow rate. Although the collection efficiency of 74.6% at 6912 L/min is relatively lower than 97% at 1535 L/min, it is still acceptable given the high flow rate. In order to meet the large space detection requirements, the flow rate is set at the maximum feasible value of 6912 L/min, which far exceeds the flow rates of existing air samplers, which are typically less than 1000 L/min [30,31,32,33]. Although it is not a technical problem to reach a higher flow rate, previous studies did not exceed the flow rate of around 1000 L/min, probably due to the decreasing collection efficiency. By combining multiple aerosol sampling strategies, including pre-charging, air–liquid interface sampling, electrostatic sampling, and turbulent deposition, the specially designed HFAS system maintained the acceptable collection efficiency of 74.6% under the 6912 L/min high flow rate. Note that since the air particle concentration declines rapidly under such a high flow rate, and the turbulence formed by this condition interferes strongly with SMPS detection, the collection efficiencies for various particle sizes have not been successfully characterized.

After optimizing the sampling voltages and flow rate, the ASMD device was validated by both artificial and environmental aerosols. In the artificial aerosol validation test, RNA solutions of varied concentrations (3 × 10^3^ and 3 × 10^5^ copies/m^3^) were prepared and aerosol particles were generated using a K5 electric sprayer. The aerosols were then collected and enriched using the ASMD device, and the sample liquid acquired from the collecting channel was delivered to different centrifuge tubes for parallel LAMP–CRISPR SAR-CoV-2 detection. The ASMD device enriched viruses from the total air in a 58 m^3^ room into 3 mL liquid sample, resulting in an enrichment ratio of 1.93 × 10^7^. As shown in Figure 3c, experimental tubes 1–5 of both 3 × 10^3^ copies/m^3^ and 3 × 10^5^ copies/m^3^ groups produced visible fluorescence signals, whereas minimal fluorescence was detected in the negative control tube 6 without target RNAs. This result demonstrated that the ASMD device is able to collect sufficient aerosols from the air and enrich target SARS-CoV-2 RNAs to levels detectable by LAMP–CRISPR. For environmental aerosol validation, the ASMD device was placed in the corridor of an apartment inhabited by SARS-CoV-2-positive patients. As shown in Figure 3d, all nine patients living in the seven rooms share the same restroom. The aerosol sampling-LAMP–CRISPR detection result is shown in Figure 3e, demonstrating that the ASMD device can perform environmental aerosol sampling for SARS-CoV-2 detection using LAMP–CRISPR. Note that the sampling time should be adjusted according to the volume of the detected space, and the 45 min procedure described in Figure S7 is used for a 58 m^3^ room. The LAMP–CRISPR results were validated by PCR and electrophoresis, as shown in Figure S9. Detailed experiment procedures are provided in the Methods section. All the above photos were taken using a camera with an optical filter, and ImageJ software (v1.54d) was used to quantify the fluorescence intensities.

In order to validate the feasibility of the MLCD chip in the ASMD device for the detection of SARS-CoV-2 aerosols, on-chip detection experiments were performed for both RNA and pseudovirus samples. For RNA samples detection, different crRNAs (S-, D614G- and N501Y-crRNAs, Table S1) were used in parallel channels on the chip to simultaneously detect different variants of the same sample. This detection experiment was repeated with various RNA samples (S-gene, D614G and N501Y). As shown in Figure 3f, for the S-gene samples, the green fluorescence intensity of the S-crRNA channel was stronger than that of the D614G- and N501Y-crRNA channels, indicating that the S-gene was successfully detected for the samples. Similarly, D614G and N501Y genes were successfully detected in D614G and N501Y samples, demonstrating that the MLCD chip can detect multiple SARS-CoV-2 variants simultaneously. For pseudovirus samples detection, on-chip LAMP–CRISPR was repeated for S-gene pseudovirus samples (>1 × 10^8^ copies/mL) using S-crRNA, and the results were compared with the S-gene RNA samples (1 × 10^9^ copies/μL). As shown in Figure 3g, both pseudovirus and RNA samples had significantly higher fluorescence intensities than the blank control, indicating positive results. Further LAMP–CRISPR experiments proved that the MLCD chip can detect a concentration as low as 10 copies/μL, which is comparable to existing automatic SARS-CoV-2 detection methods such as LAMP [33] and LAMP–CRISPR [37,43] (Figure S10). Although commercial RT-PCR-based kits have lower detection limits [47], the need for complex temperature control restricts their use in automatic detection systems. Detailed on-chip detection procedures are provided in the method section. Images were captured by a fluorescence microscope (SOPTOP, Ningbo, China) and fluorescence intensities were quantified by ImageJ software.

In summary, the above aerosol sampling and on-chip LAMP–CRISPR detection experiments demonstrate that the ASMD device is capable of effective aerosol sampling via the HFAS sampler, followed by multiplexed LAMP–CRISPR detection of SARS-CoV-2 variants via the MLCD chip.

## 4. Discussion

This study demonstrated an integrated device, ASMD, for high-flow-rate aerosol sampling and microfluidic chip-based LAMP–CRISPR detection for airborne virus surveillance. This device has the following technological advancements and prominent advantages:

1. The use of the high flow-rate aerosol sampling system, HFAS, expands the application scenarios of the device from limited room spaces to relatively large spaces, enabling long-term routine surveillance of SARS-CoV-2 aerosols in public places such as hotels or hospitals;
2. The use of a specially designed microfluidic chip, MLCD, enables visualized LAMP–CRISPR detection of multiple SARS-CoV-2 variants on a single chip, which is compatible with the aerosol sampling-detection system;
3. The combination of the aerosol sampler with the microfluidic chip leads to an all-in-one automatic aerosol sampling-SARS-CoV-2 detection system, minimizing loss of samples and reagents during the transfer between modules.

Proof-of-concept experiments on RNA, pseudovirus, and environmental samples demonstrate that the ASMD device is a feasible solution for combining high-flow-rate aerosol sampling and microfluidic chip-based LAMP–CRISPR detection for SARS-CoV-2 surveillance, enabling rapid detection of airborne SARS-CoV-2 in 85 min (45 min sampling and 40 min detection). Note that once the HFAS system’s 45 min sampling process has finished, the device initiates the detection process in the MLCD chip, while the HFAS system enters another sampling cycle. The MLCD chip, which is disposable in order to avoid sample contamination, is replaced after 40 min of detection to prepare for the next detection cycle. Thus, the ASMD device can automatically collect and monitor ambient aerosols continuously, enabling real-time warning of detected viruses in real time in a one-hour-reporting-cycle manner. For real-world application, the whole device including the chip can be stored at room temperature, although the reagents for detection need to be stored at 4 °C, and added into the system before detection. In the subsequent research, the device can be improved by the integration of a refrigerating system for reagent storage. LAMP–CRISPR detection ensured the sensitivity, specificity and stability of detection. After detection, the chip can be replaced easily, since there is a specially designed clamp for tube insertion. In conclusion, the ASMD device has a high potential for epidemic control in public spaces through airborne virus surveillance, and might be integrated into buildings’ ventilation systems for indoor aerosol surveillance and air purification in the future.

## Data Availability

The original contributions presented in this study are included in the article/Supplementary Materials. Further inquiries can be directed to the corresponding author(s).

## Supplementary Materials

The following supporting information can be downloaded at: https://www.mdpi.com/article/10.3390/bios15080475/s1, Figure S1: Dimensions of the MLCD chip; Figure S2: Fabrication process of the MLCD chip; Figure S3: Operating procedures of a single unit of the MLCD chip; Figure S4: Block diagram of the microfluidic chip-based detection system and details on the components; Figure S5: Detailed dimensions and structures of the aerosol sampling system; Figure S6: Block diagram of the HFAS system; Figure S7: Schematic diagram of the operation flow of the ASMD device for aerosol sampling and SARS-CoV-2 detection; Figure S8: Block diagram and operation interface of the automatic control module; Figure S9: Aerosol sampling PCR detection results for the generated aerosols; Figure S10: Off-chip LAMP–CRISPR detection results for different S-gene RNA concentrations; Table S1: Sequence information for S-, D614G- and N501Y-crRNA and their target sequences in the SARS-CoV-2 S gene (S, 614G, and 501Y); Table S2: Sequence information for the primers and the ssDNA reporter.

## Abbreviations

The following abbreviations are used in this manuscript:

ASMD	Microfluidic chip-based detection
HFAS	High-flow-rate aerosol sampling
MLCD	Microfluidic LAMP–CRISPR detection
SARS-CoV-2	Severe acute respiratory syndrome coronavirus 2
COVID-19	Coronavirus disease 2019
LAMP	Loop-mediated isothermal nucleic acid amplification
CRISPR	Clustered regularly interspaced short palindromic repeats
