# Supplementary material for: A Microfluidic Chip-Based Integrated Device Combining Aerosol Sampling and LAMP–CRISPR Detection for Airborne Virus Surveillance
Source: Biosensors (Basel). 2025 Jul 23;15(8):475. doi: 10.3390/bios15080475 (PMC12384654; doi:10.3390/bios15080475)
Supplement: Supplementary file 1 [file biosensors-15-00475-s001.zip › biosensors-3701728-supplementary.pdf]

Supplementary Information

A microfluidic chip-based integrated device combining aerosol sampling and LAMP-CRISPR detection for airborne virus surveillance

Anlan Zhang [1,#], Yuqing Chang [2,#], Wen Li [1,#], Yuanbao Zhang [2], Yuqian Wang [2], Haohan Xie [2], Tao Zuo [2], Yu Zhang [1], Jiyu Xi [1], Xin Wu [1], Zewen Wei [1,*], Rui Chen [2,*]

1 School of Medical Technology, Beijing Institute of Technology, Beijing 100081, China; 3120225711@bit.edu.cn (A.Z.); lwhry123@163.com (W.L.); 3120246300@bit.edu.cn (Y.Z.); 15391166971@163.com (J.X.); 3120235941@bit.edu.cn (X.W.)

2 Beijing Key Laboratory of Occupational Safety and Health, Institute of Urban Safety and Environmental Science, Beijing Academy of Science and Technology, Beijing 100054, China; m18813070133@163.com (Y.C.); zhangyuanbao2013@163.com (YB.Z.); wangyuqian1314@163.com (Y.W.); xiehaohan@iuse.ac.cn (H.X.); zuotao23@mails.ucas.ac.cn (T.Z.)

# A.Z., Y.C. and W.L. contributed equally to this work.

* Correspondence: weizewen@bit.edu.cn (Z.W.); chenrui@iuse.ac.cn (R.C.)

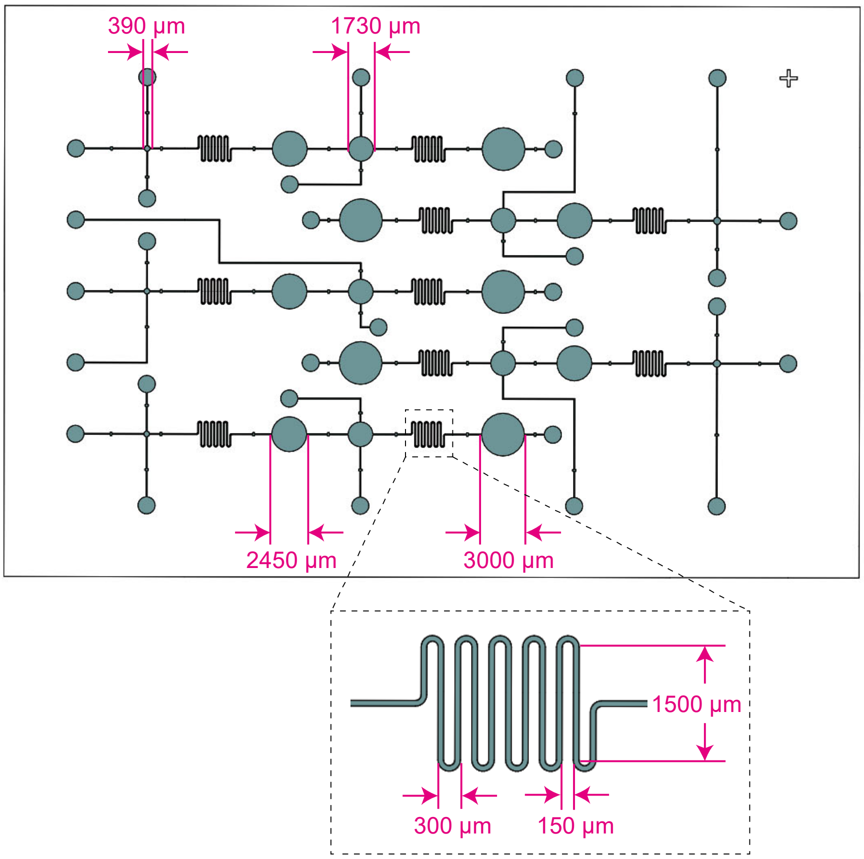

Figure S1. Dimensions of the MLCD chip. The dimensions of the MLCD chip are marked on an overlapped layout. Lower enlarged figure shows a liquid mixing channel with dimensioning.

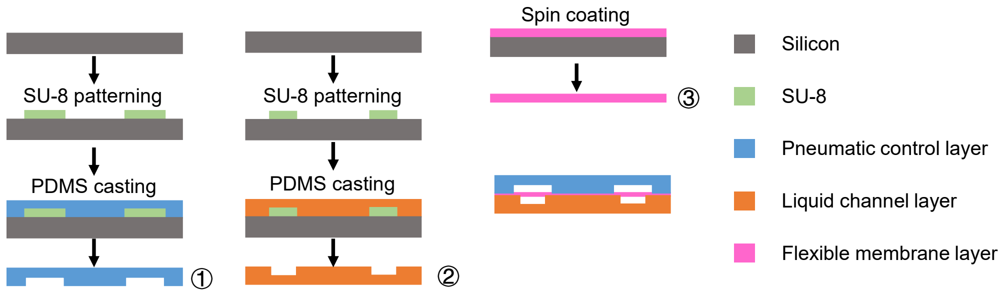

Figure S2. Fabrication process of the MLCD chip. Briefly, two polydimethylsiloxane (PDMS) layers and a flexible membrane layer were separately fabricated, and then the membrane layer and the PDMS liquid channel layer were sequentially bonded to the PDMS pneumatic control layer. The PDMS layers were fabricated by casting PDMS onto SU-8 molds which were coated and patterned on silicon wafers. The membrane layer was fabricated by spin SU-8 onto a silicon wafer.

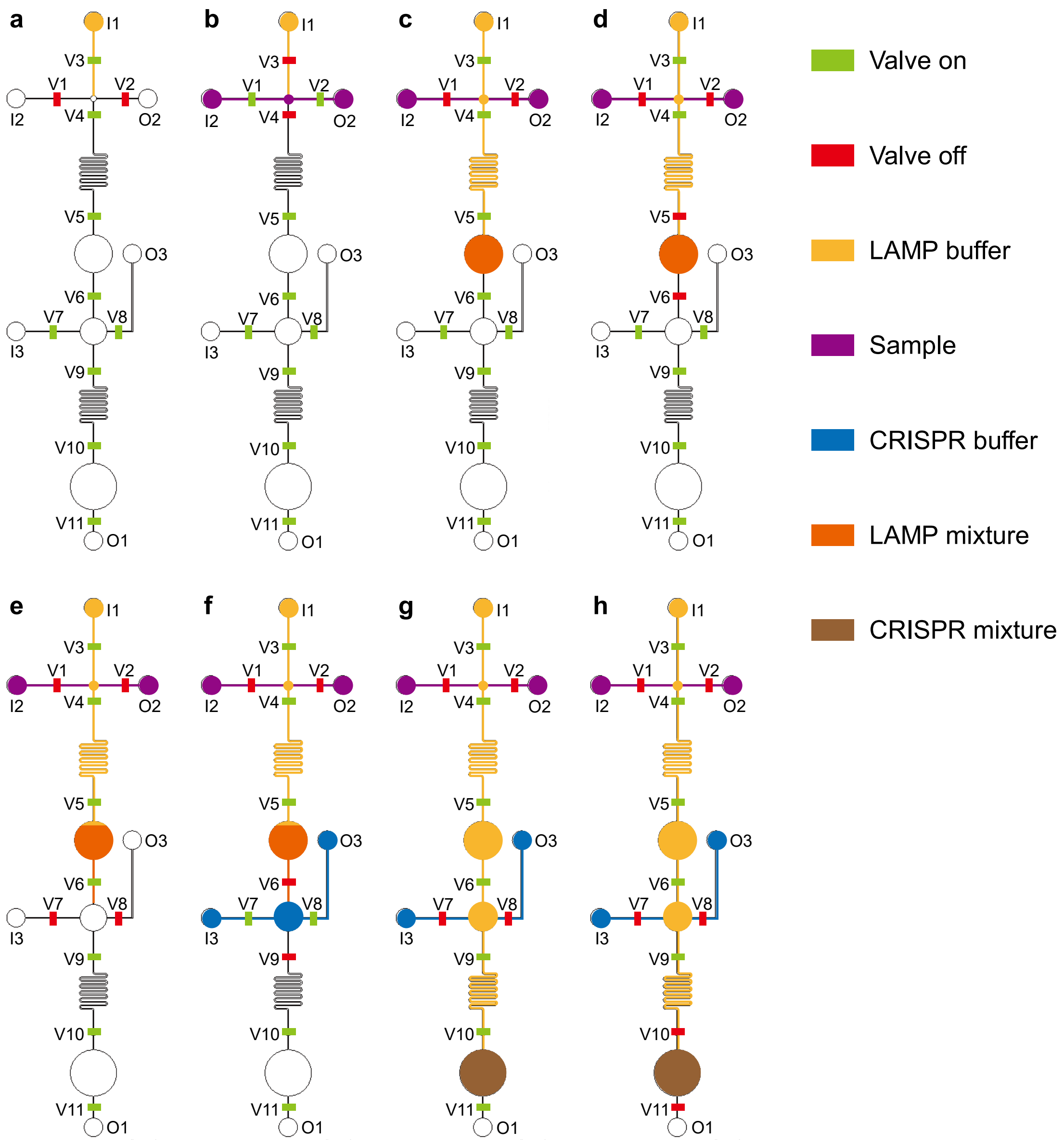

Figure S3. Operating procedures of a single unit of the MLCD chip. (**a**) Valves 1 and 2 are closed, valves 3-11 are opened. LAMP reaction buffer is injected into inlet 1 to remove air in the corresponding inlet channel. (**b**) Valves 3 and 4 are closed, valve 1 and 2 are opened. RNA sample is delivered to the RNA chamber (0.009 μL) through inlet 2. (**c**) Valves 1 and 2 are closed, valves 3 and 4 are opened. LAMP reaction buffer is injected into inlet 1 and carries sample to the LAMP reaction chamber (0.36 μL). (**d**) Valves 5 and 6 are closed. The chip is heated at 65 °C for 40 min to perform LAMP amplification. (**e**) Valves 7 and 8 are closed, valves 5 and 6 are opened. Liquid is injected into inlet 1 to push LAMP reaction mixture into the subsequent channel, in order to remove air. (**f**) Valves 6 and 9 are closed, valves 7 and 8 are opened. CRISPR reaction buffer is delivered to the buffer chamber (0.18 μL) through inlet 3. (**g**) Valves 7 and 8 are closed, valves 6 and 9 are opened. Liquid is injected into inlet 1 to push LAMP reaction mixture and CRISPR reaction buffer to the CRISPR detection

chamber (0.54 μL). (**h**) Valves 10 and 11 are closed. The chip is heated at 37 ℃ for 5 min to perform CRISPR reaction.

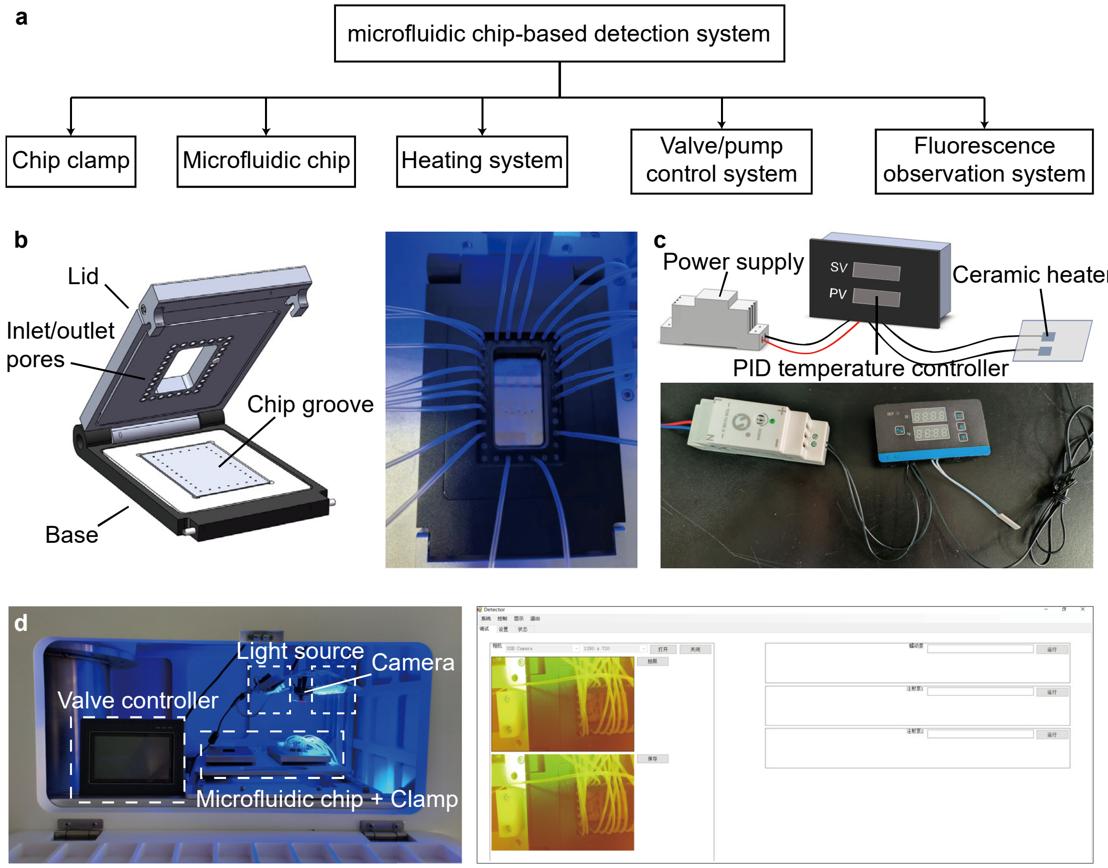

Figure S4. Block diagram of the microfluidic chip-based detection system (**a**) and details on the components. (**b**) To ensure stable flow of liquid/gas and stable visualization of detection result, and to simplify the procedure of replacing the chip, a clamp has been designed to fix the chip. (**c**) A heating system has been designed for on-chip LAMP amplification (65 °C) and CRISPR detection (37 °C). The system consists of a ceramic heater, a PID smart temperature controller and a guide rail type switching power supply. The temperature probe of the PID controller detects the temperature of the chip chambers and sends the signal to the controller. The PID controller then adjusts the on/off state of the ceramic heater in real time according to the temperature feedback, keeping the temperature at the set value. Since the PID output temperature directly controls the ceramic heater, and is higher than the chip's temperature, the output temperatures for LAMP (65 °C) and CRISPR (37 °C) reactions are tested and set at 80 and 40 °C, respectively. (**d**) The fluorescence observation system consists of a blue light source (480 nm) and a color filter camera and outputs the visualized result on the computer software interface in real time.

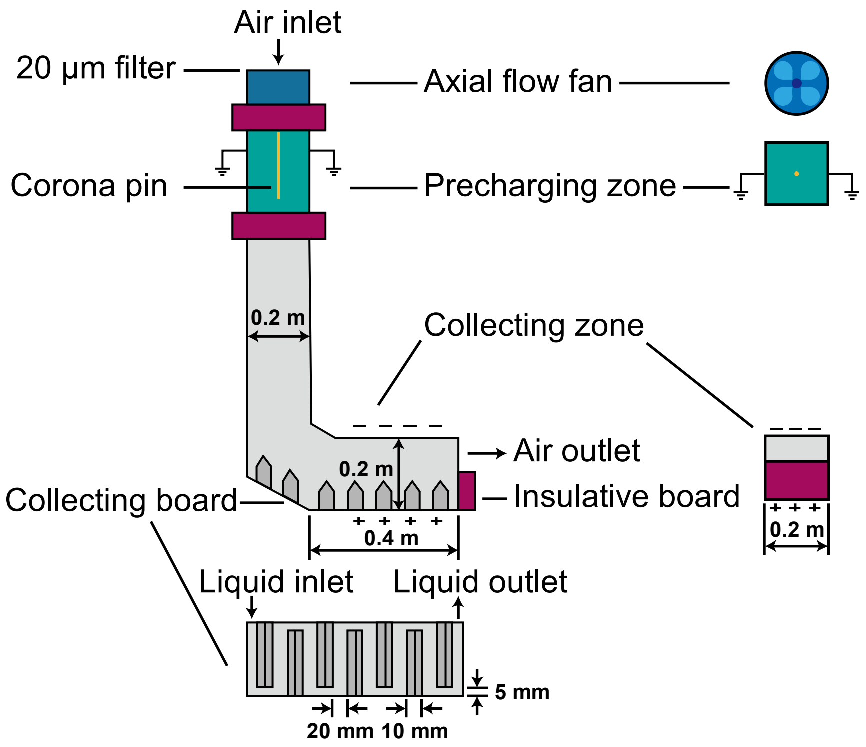

Figure S5. Detailed dimensions and structures of the aerosol sampling system. Air drawn in by the axial flow fan (235.5 CFM, 0-6912 L/min; ZHJ26-172-RD , Yiheda, China) is filtered through a 20 μm filter before going to the precharging zone. A corona pin is placed at the center of the precharging zone, in order to provide the precharging voltage together with a DC power supply (0-60 kV; TCM6002, Telsaman, China) set outside the precharging zone. The side length of the collecting zone's square cross section is set to 0.2 m to fulfill the requirement for high flow rate air intake. The length of the collecting zone is set to 0.4 m, in order to contain a long zigzag collecting channel for air-liquid interface sampling under the collecting voltage provided by an outside DC power supply (0-60 kV; TCM6002, Telsaman, China). The collecting board with the zigzag channel has a liquid inlet for the infusion of lysis buffer and magnetic beads.

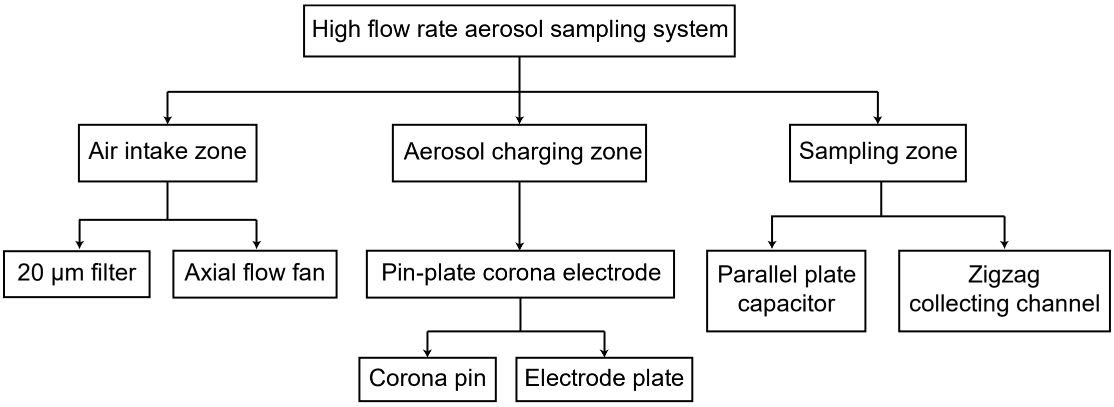

Figure S6. Block diagram of the HFAS system.

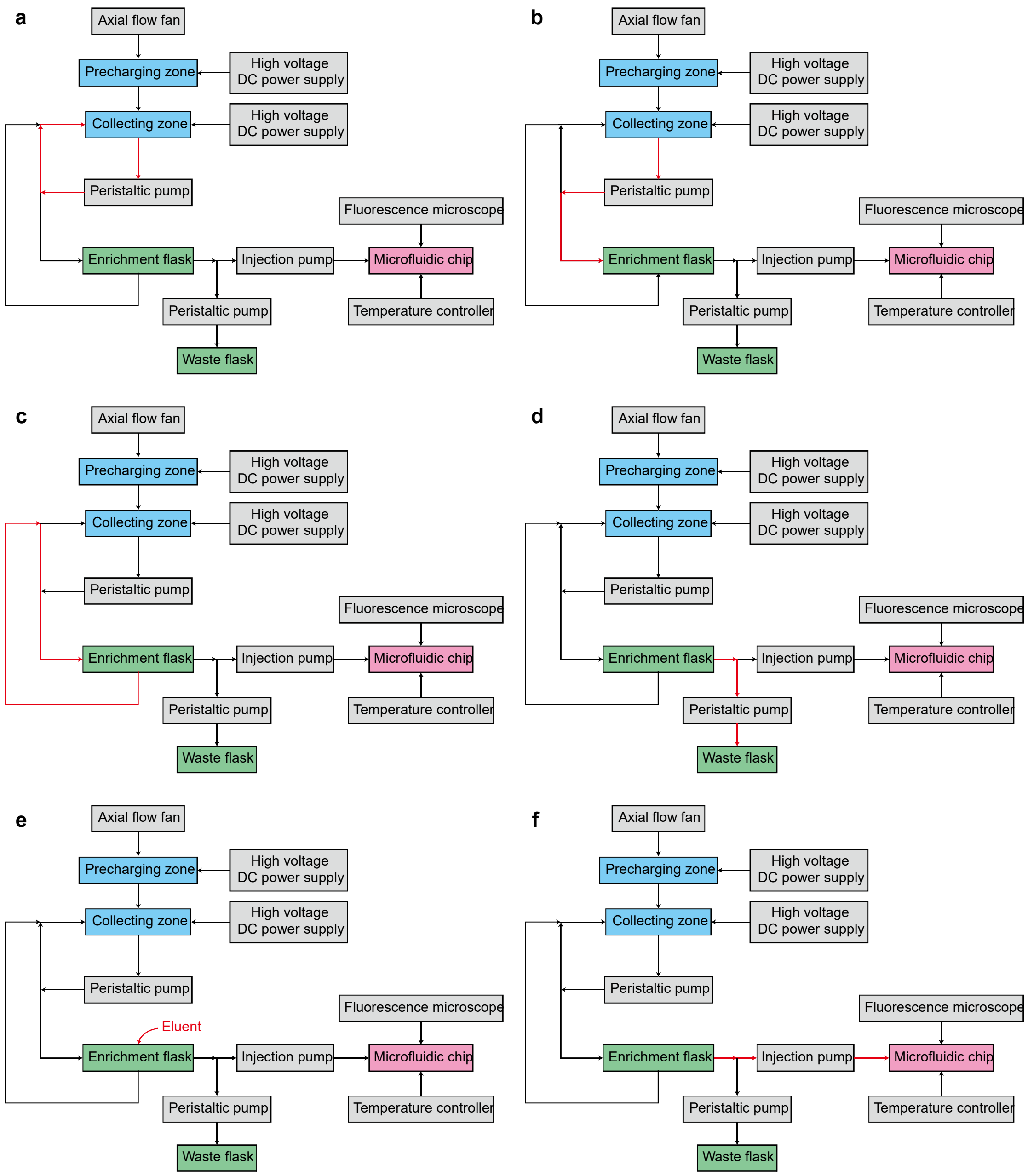

Figure S7. Schematic diagram of the operation flow of the ASMD device for aerosol sampling and SARS-Cov-2 detection. The ASMD device operates in three steps: Aerosol particle collection and RNA extraction, RNA enrichment, and on-chip LAMP-CRISPR detection. For aerosol particle collection and RNA extraction, air drawn in by the axial flow fan and precharged in the precharging zone cycles in the collecting zone for 30 min, in order to collect aerosol particles into the sampling liquid, which contains lysis buffer and magnetic beads to lyse the aerosol particles and extract the released RNAs (**a**). For RNA enrichment, first, the sample liquid containing RNA-binding magnetic beads is delivered to the enrichment flask for 2 min, and the RNA-beads are adsorbed to the flask wall by the surrounding magnet (**b**). Next, the sample liquid cycles in and out of the enrichment flask for 10 min to

fully adsorb the RNA-beads (**c**). Then, the rest of the liquid is delivered to the waste flask (**d**). At last, eluant is injected into the enrichment flask and let stand for 1 min to elute target RNAs from the magnetic beads (**e**). After enrichment, sample liquid containing the eluted RNAs is delivered to the microfluidic chip for 40 min LAMP-CRISPR detection (**f**).

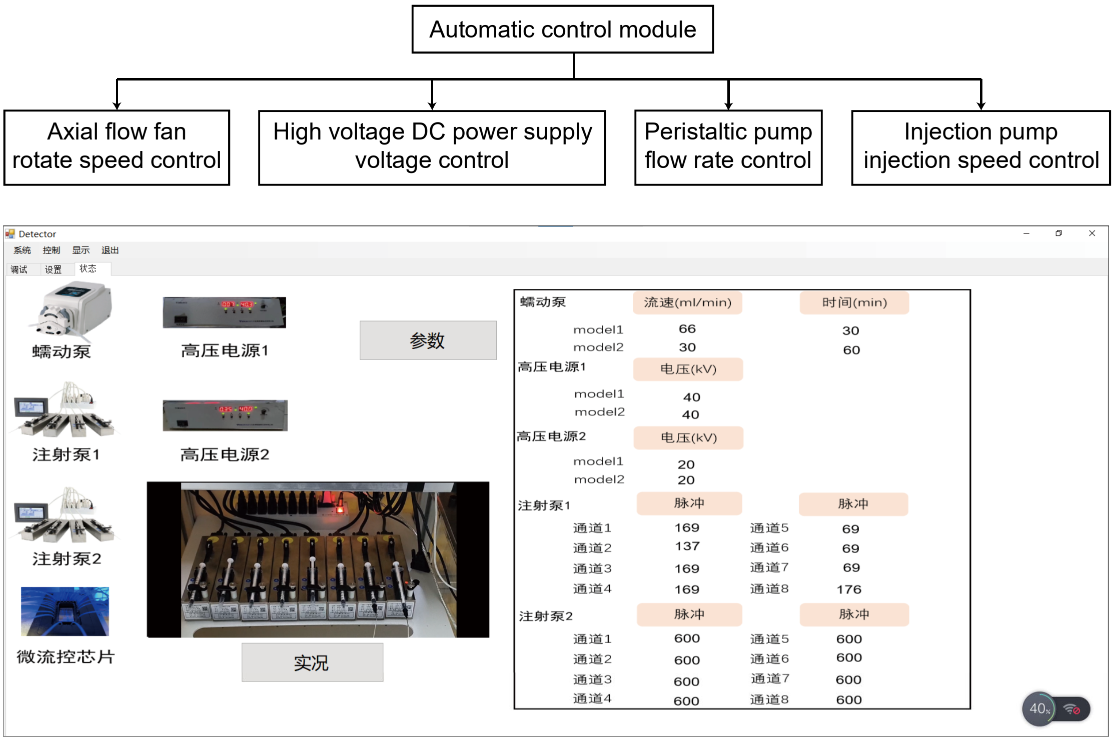

Figure S8. Block diagram and operation interface of the automatic control module.

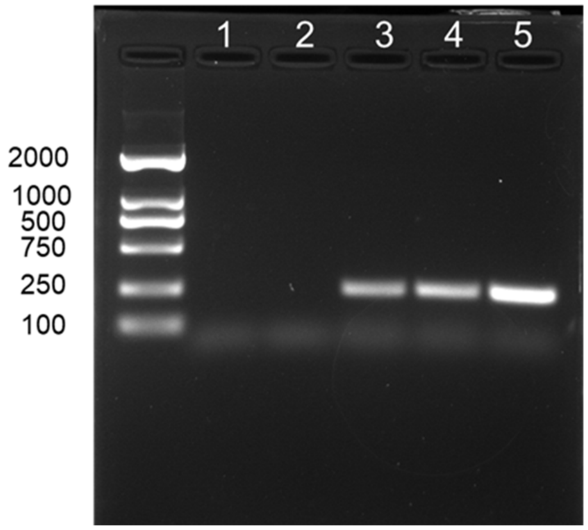

Figure S9. Aerosol sampling-PCR detection result for the generated aerosols. 2 different concentrations of RNA solution (low concentration: 2 μL of 90 ng/μL RNA diluted to 380 mL resulting in 0.47 ng/mL; high concentration: 10 μL of 90 ng/μL RNA diluted to 380 mL resulting in 2.37 ng/mL) were used to generate the aerosols. Electrophoretic bands 1 and 2 were the results of low and high concentration groups without magnetic bead enrichment. Electrophoretic bands 3 and 4 were the results of low and high concentration groups after enrichment, respectively. Electrophoretic band 5 was the result of the 90 ng/μL RNA solution. As can be seen, the enrichment step was necessary for the successful detection of both low and high concentrations of RNA aerosols, and the concentrations of the collected samples correspond to the concentrations of the aerosols.

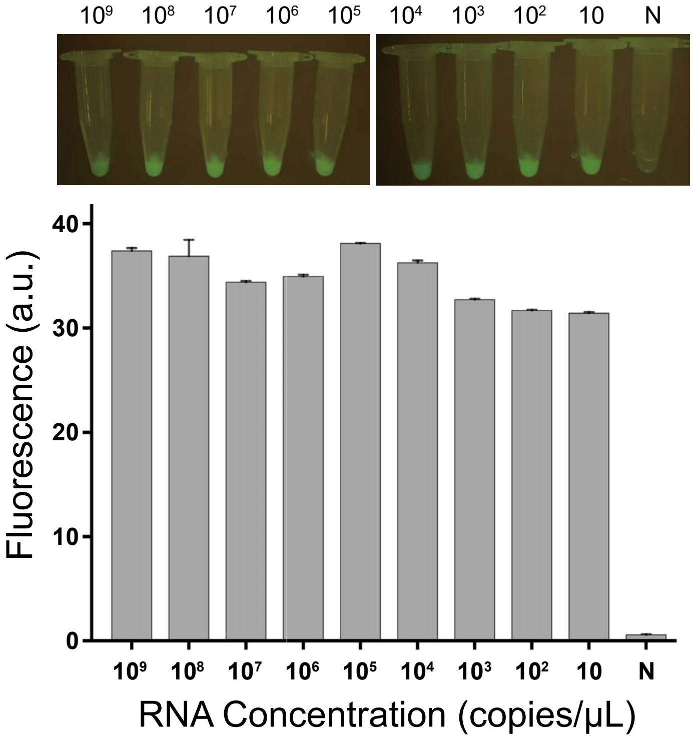

Figure S10. Off-chip LAMP-CRISPR detection result for different S-gene RNA concentrations.

Table S1. Sequence information for S-, D614G- and N501Y-crRNA and their target sequences in the SARS-Cov-2 S gene (S, 614G and 501Y).

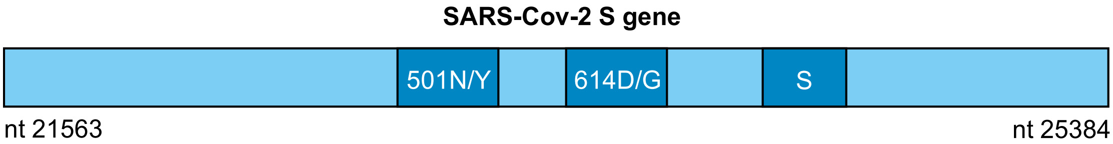

Sequence name	Sequence (5'-3')
S	AGCTTCTGCTAATCTTGCTGCTAC
S-crRNA	UCGAAGACGAUUAGAACGACGAUGUAGAUGUUGUCAUCUUUAAG
614D	TTTATCAGGATGTTAACTGCAC
614G	TTTATCAGGGTGTTAACTGCAC
D614G-crRNA	UAAUUUCUACUCUUGUAGAUUCAGGGUAUUAACUGCACAGAAG
501N	TTTCCAACCCACTAATGGTGTT
501Y	TTTCCAACCCACTTATGGTGTT
N501Y-crRNA	UAAUUUCUACUAAGUGUAGAUCAACCCACUUAUGGUGTTGGTTAC

Table S2. Sequence information for the primers and the ssDNA reporter.

Gene	Primer name	Primer sequence (5'-3')
S gene	T7-FP-1	GAAATTAATACGACTCACTATAGGGTGGTCAACC-AAAATGCACA
	T7-RP-1	TGATTTGTGGTTCATAAAAATTCC
	PCR-F	ATGATATCCTTTCACGTCTTGACAAAGTTGAGG
	PCR-R	GAAGGACATAAGATGATAGCCCTTTCCACAAAAA
	FIP	GCTATCATCTTATGTCCTTCCCTCACATAAGTCAC-ATGCAAGAAGA
	BIP	ACACTCTGACATTTTAGTAGCAGCGTGACTCAAC-AATTAATTAGAGC
	F3	TGTGAAGTTCTTTTCTTGTGC
	B3	GACTTCAAAGTTTGCAGACA
	LF	AAGATTAGCAGAAGCTCTGATT
	LB	GTCAGCACCTCATGGTGTAG
D614G	T7-FP-2	TAATACGACTCACTATAGGGAGGGTTTTCCCAGT-CACG
	T7-RP-2	GAGCGGATAACAATTTCACAC
	FIP	GACACCACCAAAAGAACATGGTGGCTGACACTA-CTGATGCTG
	BIP	TCCCTGTTGCTATTCATGCAGATGTTTGAAAAAC-ATTAGAACCTGTAG
	F3	AACAATTTGGCAGAGACATT
	B3	TGAGTTGTTGACATGTTCAG
	LF	CTCAAGTGTCTGTGGATCACGG
	LB	CAACTTACTCCTACTTGGCGTG
N501Y	FIP	TCAACACCATTACAAGGTGTGCTATAATCTCAAA-CCTTTTGAGAGAG
	BIP	TACAATCATATGGTTTCCAACCCAAGAAAGTACT-ACTACTCT GTATGG
	F3	CCTGTATAGATTGTTTAGGAAGTC
	B3	TGGTGCATGTAGAAGTTCAA
	LF	CCGGCCTGATAGATTTCAGTTG
ssDNA reporter		6-FAM-TTATT-BHQ1
